# Effects of temperature, soil substrate, and microbial community on carbon mineralization across three climatically contrasting forest sites

**DOI:** 10.1002/ece3.3708

**Published:** 2017-12-10

**Authors:** Zuoxin Tang, Xiaolu Sun, Zhongkui Luo, Nianpeng He, Osbert Jianxin Sun

**Affiliations:** ^1^ College of Forest Science Beijing Forestry University Beijing China; ^2^ CSIRO Agriculture & Food Canberra Australia; ^3^ Key Laboratory of Ecosystem Network Observation and Modeling Institute of Geographic Sciences and Natural Resources Research Chinese Academy of Sciences Beijing China

**Keywords:** decomposition, forest, inoculation, microorganism, mineralization, soil organic matter, thermal adaptation

## Abstract

How biotic and abiotic factors influence soil carbon (C) mineralization rate (*R*_S_) has recently emerged as one of the focal interests in ecological studies. To determine the relative effects of temperature, soil substrate and microbial community on *R*_s_, we conducted a laboratory experiment involving reciprocal microbial inoculations of three zonal forest soils, and measured *R*_S_ over a 61‐day period at three temperatures (5, 15, and 25°C). Results show that both *R*_s_ and the cumulative emission of C (*R*_cum_), normalized to per unit soil organic C (SOC), were significantly affected by incubation temperature, soil substrate, microbial inoculum treatment, and their interactions (*p *<* *.05). Overall, the incubation temperature had the strongest effect on the *R*_S_; at given temperatures, soil substrate, microbial inoculum treatment, and their interaction all significantly affected both *R*_s_ (*p *<* *.001) and *R*_cum_ (*p *≤* *.01), but the effect of soil substrate was much stronger than others. There was no consistent pattern of thermal adaptation in microbial decomposition of SOC in the reciprocal inoculations. Moreover, when different sources of microbial inocula were introduced to the same soil substrate, the microbial community structure converged with incubation without altering the overall soil enzyme activities; when different types of soil substrate were inoculated with the same sources of microbial inocula, both the microbial community structure and soil enzyme activities diverged. Overall, temperature plays a predominant role in affecting *R*_s_ and *R*_cum_, while soil substrate determines the mineralizable SOC under given conditions. The role of microbial community in driving SOC mineralization is weaker than that of climate and soil substrate, because soil microbial community is both affected, and adapts to, climatic factors and soil matrix.

## 1 INTRODUCTION

Microbial decomposition of soil organic matter (SOM) is the core process of soil carbon (C) mineralization and nutrient cycling, linking closely to other ecosystem functionalities (Bardgett & van der Putten, 2014; van der Heijden, Bardgett, & Van Straalen, 2008). Understanding the roles and underlying mechanisms of soil microbial communities in driving SOM decomposition is critical for modelling the terrestrial carbon cycling in the context of global climate change and environmental perturbations (Bardgett, Freeman, & Ostle, 2008; Schmidt et al., 2011; Xu et al., 2014).

Soil microorganisms may adapt to varying soil matrix including a complex array of substrates, physiochemical conditions, and biotic interactions; alteration in the soil matrix, in turn, may modify microbial community structure and activity, hence SOM decomposition and stability (Schimel & Schaeffer, 2012; Strickland, Lauber, Fierer, & Bradford, 2009; Sun, Zhao, You, & Sun, 2016; You et al., 2014). In the organic layer of soil profile, however, differences in microbial community composition, size, and physiology may affect the rate and trajectory of carbon mineralization as a result of differential functionalities among contrasting microbial community types (Keiluweit et al., 2015; Waldrop & Firestone, 2004).

It is well documented that both climate and vegetation types exert significant impacts on soil C dynamics as well as microbial community structures (Brockett, Prescott, & Grayston, 2012; Cong et al., 2015; Hackl, Pfeffer, Donat, Bachmann, & Zechmeister‐Boltenstern, 2005). Climatic differences, in particular temperature and precipitation, can often explain the largest proportion of variations in SOM decomposition at regional and global scales (Carvalhais et al., 2014; Sun, Campbell, Law, & Wolf, 2004); this climate–SOM relationship is widely adopted in ecosystem C cycle models (McGuire & Treseder, 2010; Xu et al., 2014). Vegetation type may determine the size of soil C pool and microbial community structure through direct effects of the quantity and quality of detritus inputs and indirect effects of modification of soil physiochemical and properties (Prescott & Grayston, 2013; Toriyama, Hak, Imaya, Hirai, & Kiyono, 2015; Wan et al., 2015; You et al., 2014). Therefore, soils under different climatic conditions and vegetation types may differ in microbial community structure (Drenovsky, Steenwerth, Jackson, & Scow, 2010; Foesel et al., 2014), and consequently the microbial functional activities and C utilization (He et al., 2013; Reinsch et al., 2013). However, it is still unclear what would be the combined effects and the relative contributions of microbial community and soil properties on *R*_s_ among sites with large differences in temperature and precipitation.

In this study, we conducted a fully reciprocal incubation experiment to determine the relative effects of soil physiochemical properties and microbial communities on *R*_s_, involving soil substrate and soil inocula originated from cool temperate, warm temperate, and subtropical forests. The incubation was carried out concurrently at three temperatures (5, 15, and 25°C) and constant soil moisture. *R*_s_ was repeatedly measured during incubation over 61 days at regular intervals. Selective soil and microbial variables were also determined at start and/or end of the incubation. Using the collected datasets, we aim to address two questions: (i) Which of soil physiochemical properties and microbial community is more important in determining SOC mineralization? and (ii) do soils of different climatic originals differ in thermal adaptation of SOC decomposition?

## 2 MATERIAL AND METHODS

### 2.1 Sites and field sampling

The incubated soils were collected from three zonal forests with contrasting climatic conditions, including a cool temperate forest in the Changbai Mountains National Nature Reserve (Changbai), a warm temperate forest (WT) in the Baotianman National Nature Reserve (Baotianman), and a subtropical forest (ST) in the Dinghu Mountains National Nature Reserve (Dinghu). Basic information on these sites, soil properties, and vegetation is summarized in Table 1.

**Table 1:** Selective information of sites, soil characteristics of 0–10 cm depth and vegetation

Variables	Sites		
	Changbai (cool temperate forest, CT)	Baotianman (warm temperate forest, WT)	Dinghu (subtropical forest, ST)
Latitude	42°23′24″–24′33″N	33°29′30″–31′2″N	23°09′21″–11′30″N
Longitude	128°05′11″–06′5″E	111°55′51″–56′12″E	112°30′39″–33′41″E
Annual rainfall (mm)	700–1,400	900	1927
Mean annual air temperature (°C)	3–7	15.1	21.4
Soil pH	5.8	4.56	4.04
Soil clay content (%)	11.9	11.7	10.8
Total soil C (g C kg^−1^ soil)	131.1	42.3	48.7
Total soil N (g N kg^−1^ soil)	9.13	2.60	2.30
Microbial biomass C (mg C kg^−1^ soil)	608.8	176.8	427.8
Microbial biomass N (mg N kg^−1^ soil)	59.2	12.9	77.2
Total PLFAs (nmol g^−1^ soil)	70.2	71.4	19.5
Forest type	Mixed broad‐leaved/Korean pine forest	Mixed pine/oak forest	Mixed pine/broad‐leaved forest
Dominant plant species	*Pinus koraiensis* Sieb. et Zucc.*Tilia amurensi* Rupr*Acer pictum* subsp. *mono* (Maxim.)*Fraxinus mandschurica* Rupr.	*Pinus armandii* Franch*Quercus aliena* var. *acuteserrata* Maxim.	*Pinus massoniana* Lamb.*Schima superba* Gardn. et Champ.*Castanea henryi* (Skan) Rehd. et Wils.
Stand age (years)	170–300	55–65	75–85
Soil type	Mountainous dark brown forest soil	Dystric cambisols	Lateritic red soil

Field sampling was conducted from May to June of 2013. In each forest, we first set up three 20 m × 20 m plots spatially separated. In each plot, 24 soil cores were collected using a stainless‐steel soil sampler (3‐cm inner‐diameter) to a depth of 10 cm. All soil samples in each forest were mixed to form a single composite sample, which was then placed in sealed bags and stored in an ice cooler within 2 hr of collections. Gravels, roots, and large organic residues were manually removed before passing a 2‐mm sieve. In the laboratory, samples were divided into three parts: One was stored at −20°C for analyses of soil enzyme activities, microbial community composition. The second part was used for the incubation experiment, in which majority of the soils were sterilized by autoclaving and used as soil substrate; a small proportion remained unsterilized and used as microbial inoculum. The third part was used to measure soil water‐holding capacity (WHC, %), soil gravimetric moisture (%), and soil properties (e.g., C, N, and pH).

### 2.2 Experimental design and treatments

The experiment was set up as a full factorial arrangement consisting of three soil substrates (soils of CT, WT, and ST), three microbial inoculum sources (CT inoculum, WT inoculum, and ST inoculum), and three incubation temperatures (5, 15 and 25°C), with five replications.

The soils used as substrate were treated with autoclaving (121°C, 45 min) twice in succession and again 24 hr later for complete sterilization (Nie et al., 2013). The method of autoclaving was to maximize the chance for microbial communities being introduced only via the inoculum (Fanin & Bertrand, 2016). Soil inocula were prepared as freshly sieved soil through a 1‐mm mesh screen without sterilization (van de Voorde, van der Putten, & Bezemer, 2012), with microbial biomass content of 70 nmol/g dry soil for the CT inoculum, 71 nmol/g dry soil for the BT inoculum, and 19 nmol/g dry soil for the DH inoculum.

Following the final autoclaving, the soil substrates were placed in 150‐ml sterilized plastic bottles (24 g fresh weight of soil substrate to a bottle), with all the tools that used to weigh the soil substrate and the plastic bottles sterilized and the processes conducted in a super clean bench in the laboratory to avoid contamination. All the bottles were preincubated at designated temperatures (5, 15 and 25°C, respectively) for 4 days to assess the effectiveness of sterilization. Soil C mineralization rate (*R*_S_) was measured during the period of preincubation. There were very little activities detected (average 0.036–0.077 μg CO_2_ g^−1^ soil day^−1^, representing only 0.6%–1.3% of the microbial respiration in substrate soil without autoclaving), likely as a result of abiotic CO_2_ production, extracellular enzyme activities or remnant microbial populations (Nie et al., 2013).

After the preincubation, soil inocula were introduced into each of the bottles filled with soil substrate specimen, as a 6:1 mixture of soil substrate and the inoculum (Nie et al., 2013; van de Voorde et al., 2012). Three bottles containing autoclaved soil samples without addition of microbial inoculum (three replicates) were used as controls for each soil substrate and each incubation temperature over the entire incubation period. All the specimen bottles were incubated at designated temperatures for a period of 61 days. During incubation, the moisture in all specimen bottles was maintained at 50% of water‐holding capacity (Strickland et al., 2009) by repeatedly weighing and adjusting water.

### 2.3 Measurements of soil physiochemical properties and *R*_s_

We measured soil pH, SOC, total nitrogen (TN), particle size distribution, effective metal ions (Fe, Cu, and Mn), and microbial biomass C and nitrogen (MBC and MBN) of the bulk soils, both before and after the incubation experiment. Soil pH was measured by mixing the soil sample with deionized water at a 1:2.5 ratio (w/v). The supernatants were measured with a pH meter (HI‐9125, Hanna Instruments Inc, Woonsocket, RI). SOC content was measured by a K_2_Cr_2_O_7_–H_2_SO_4_ calefaction method (Nelson & Sommers, 1982), and TN by a Kjeldahl digestion procedure (Gallaher, Weldon, & Boswell, 1976). Particle size distribution was determined as percentage of sand (>53 μm), coarse slit (20–53 μm), fine silt (2–20 μm), and clay (<2 μm), using the sifter and centrifugal method (Gee, Bauder, & Klute, 1986). The effective Fe, Cu, and Mn were measured by inductively coupled plasma‐atomic emission spectrometry (ICP‐AES; Li, Coles, Ramsey, & Thornton, 1995). MBC and MBN were measured by the fumigation‐extraction method (Vance, Brookes, & Jenkinson, 1987).

*R*_s_ was measured 13 times using an Automatic Temperature Control Soil Flux System (PRI‐8800; Pri‐Eco, Beijing, China) as described in He et al. (2013); this system has been successfully used in studies of Wang et al. (2016), Liu et al. (2016), and Li et al. (2017). We calculated *R*_s_ for day 0, 1, 2, 4, 6, 9, 13, 19, 32, 39, 45, 52, and 61 of the incubation. The system samples and measures the rate of soil respiration at programed time intervals automatically.

In practice, *R*_s_ was calculated from the slope of the CO_2_ concentration as (He et al., 2013):

(1) $$R_{s}=\frac{A\times V\times \alpha \times \beta }{M}$$

where *R*_s_ is soil C mineralization rate, *A* the slope of the CO_2_ concentration in bottle, *V* the volume of the specimen bottle and gas tube, *M* the weight of soil specimen, α the transformation coefficient of CO_2_ mass, and β the transformation coefficient of time. We calculated the daily *R*_s_ (μg CO_2_‐C g^−1^ soil day^−1^). On each measurement date, the daily *R*_s_ was adjusted against the controls. The mean daily *R*_s_ (μg CO_2_‐C g^−1^ SOC day^−1^) and the cumulative C mineralization (*R*_cum_, μg CO_2_‐C g^−1^ SOC) over the 61‐day incubation period were normalized to per unit SOC.

### 2.4 Measurements of soil enzyme activity and microbial community composition

Measurements were taken on the activities of selective soil extracellular enzymes, and soil microbial community composition prior to and after the incubation experiment. We measured the activities of four soil enzymes that are involved in degrading lignin (phenol oxidase, PO and peroxidase, PER), cellulose (β‐1,4‐glucosidase, BG), and chitin (*N*‐acetyl‐β‐glucosaminidase, NAG), respectively (You et al., 2014, 2016). PO and PER were measured using 1‐3,4‐dihydroxyphenyla‐lanine (L‐DOPA) as substrate (Li et al., 2010; Sinsabaugh et al., 1993). For phenol oxidase, the reaction mixture was composed of 2 ml of 5 mmol L^−1^ L^−1^ L‐DOPA solution and soil slurry (1 g fresh soil with 1.5 ml 50 mmol L^−1^ L^−1^ sodium acetate buffer), and peroxidase activity assays received 2 ml of 5 mmol L^−1^ L^−1^ L‐DOPA and soil slurry (1 g fresh soil with 1.5 ml 50 mmol L^−1^ L^−1^ sodium acetate buffer), plus 0.2 ml of 0.3% H_2_O_2_. The activities of BG and NAG were determined by the conventional β‐nitrophenol assays (Baldrian, 2009; Parham & Deng, 2000). All enzyme activities were calculated on per unit of SOC basis (You et al., 2016).

Soil microbial community composition was analyzed using the phospholipid fatty acid (PLFA) method following Bossio and Scow (1998). Concentrations of individual PLFAs were calculated based on 19:0 internal standard concentrations; the samples were analyzed on a MIDI Sherlock microbial identification system 6.0 (microbial ID, Inc. Newark, DE 19713). The indicator PLFAs were used for classification of microbial community types. Bacterial community (*B*) was considered to be comprised of PLFAs i14:0, 15:0, i15:0, a15:0, i16:0, 16:1w7c, 17:0, a17:0, cy17:0, 18:1w7c and cy19:0; gram‐positive bacteria (*PB*) of i14:0, i15:0, a15:0, i16:0, a17:0, i17:0; gram‐negative bacteria (*NB*) of 16:1w7c, cy17:0, 18:1w7c, cy19:0; actinomycete (*Act*) of 10Me16:0, 10Me17:0 and 10Me18:0; saprotrophic fungi (*Sap*) of 18:2w6,9c; and arbuscular mycorrhizal fungi (*F*) of 16:1w5c. Other PLFAs (*other*) such as 14:0, 16:0, 16:1 w9c, 17:1w8c, and 18:1w9c were also used for analysis of the microbial community (You et al., 2014, 2016).

### 2.5 Data analysis

We used the Kruskal's dissimilarity matrices (Kruskal, 1964) to discriminate the soil properties, microbial community structure, and microbial enzyme activity of soil samples in different forests. The soil properties integrate physiochemical variables including soil pH, SOC, TN, soil C:N ratio, MBC, MBN, microbial biomass C:N ratio, soil particle size distribution, and effective metal ions (Cu, Fe, and Mn). For microbial community structure, we included all individual PLFAs in the analysis. The microbial enzyme activity is represented by the activities of the four soil extracellular enzymes determined in this study, that is, PO, PER, BG, and NAG.

The effects of soil substrate, microbial inocula, and incubation temperatures on *R*_s_ were tested by repeated measures ANOVAs with measurement time as a covariate for the full experimental period of 61 days as well as for two contrasting periods of 0–32 days (representing a period of microbial colonization and active C mineralization) and 39–61 days (representing a period of settled microbial community and constrained C mineralization). A full factorial ANOVA including all treatment factors as well as separate ANOVAs by incubation temperatures was performed to examine the effects on *R*_cum_. Duncan's multiple‐rang test was used to separate differences among means at the level of *p *<* *.05. These statistical analyses were performed by SPSS (version 17.0).

The Kruskal's dissimilarity matrices were also used to compare the microbial community structure and microbial enzyme activity between at the start and at the end of the incubation experiment.

To determine how microbial community structure and microbial enzyme activity vary among the three soils reciprocally treated with inocula of different origins, we conducted a Principle Component Analysis (PCA) at the end of the incubation. Rather than using individual PLFAs to indicate microbial community structure, we used seven categorized microbial community groups (i.e., *B*,* PB*,* NB*,* Act*,* Sap*,* F*, and *other*) in the PCA. We also determined the linkage between soil microbial community groups and the activities of four extracellular enzymes in the PCA. These analyses were conducted using R 3.0.2.

## 3 RESULTS

### 3.1 Differences in soil‐related characteristics in three zonal forests

**Figure 1:**
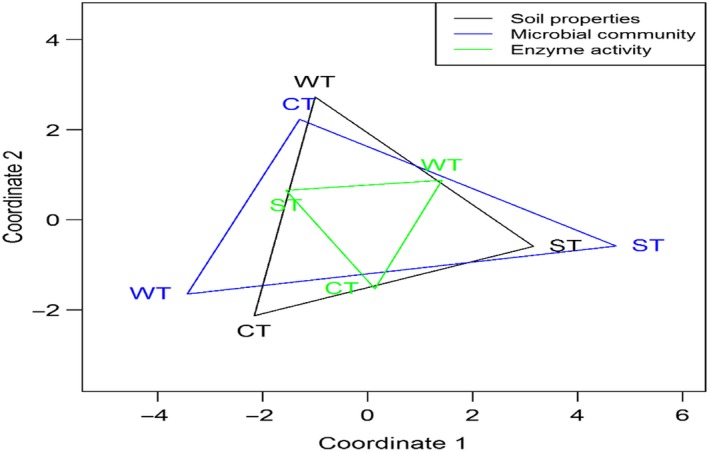
Kruskal's dissimilarity matrices (nonmetric multidimensional scaling) illustrating the dissimilarities in soil properties, microbial community structure, and microbial enzyme activities among the soils of the cool temperate (CT), warm temperate (WT), and subtropical (ST) forests. Greater distance between apexes of the triangle indicates greater dissimilarity between two soils. The area of the triangle demonstrates the overall dissimilarity between the soils

There were clear distinctions in original characteristics, notably the soil properties and the microbial community structure in the three zonal forests (Figure 1). The greatest differentiation was observed between the WT and ST soils in the microbial community structure. Among the three categories of soil‐related characteristics, the microbial enzyme activity was least discriminated among the three soils (Figure 1).

### 3.2 Changes in soil C mineralization and temperature sensitivity

The treatment factors and interactions all had highly significant effects (*p *<* *.001) on the daily *R*_s_ and significant effects (*p *<* *.05) on *R*_cum_ (Table 2). The daily *R*_s_ was most strongly affected by the incubation temperature (*F *=* *998.5), followed by the soil substrate (*F *=* *482.4). However, for *R*_cum_, the soil substrate had the greatest effect (*F *=* *243.8), followed by the incubation temperature (*F *=* *127.6). The effects of the microbial inoculum on both *R*_s_ and *R*_cum_ were much weaker compared with the other two main factors, albeit statistically also highly significant (*p *<* *.001; Table 2).

**Table 2:** Summary of full ANOVAs for testing the treatment effects daily C mineralization rate (*R*_s_) and the cumulative C mineralization (*R*_cum_) during 0–32, 33–61 and 0–61 days over a 61‐day incubation period

Factors	Daily *R*_s_ (0–32 days)			Daily *R*_s_ (33–61 days)			Daily *R*_s_ (0–61 days)			*R*_cum_ (0–61 days)		
	*df*	*F*	*p*	*df*	*F*	*p*	*df*	*F*	*p*	*df*	*F*	*p*
Soil substrate (soil)	2	432.8	<.001	2	137.8	<.001	2	482.4	<.001	2	243.7	<.001
Microbial inoculum (Micr)	2	17.2	<.001	2	53.7	<.001	2	24.34	<.001	2	26.1	<.001
Temperature (Temp)	2	1033.4	<.001	2	59.9	<.001	2	998.54	<.001	2	127.6	<.001
Soil × Micr	4	21.3	<.001	4	24.5	<.001	4	26.3	<.001	4	21.4	<.001
Soil × Temp	4	40.3	<.001	4	43.9	<.001	4	36.0	<.001	4	2.91	.026
Micr × Temp	4	19.5	<.001	4	10.4	<.001	4	22.2	<.001	4	14.2	<.001
Soil × Micr × Temp	8	30.7	<.001	8	16.7	<.001	8	34.5	<.001	8	20.3	<.001
Error	93	—	—	93	—	—	93	—	—	120	—	—

CT, cool temperate forest; WT, warm temperate forest; ST, subtropical forest.

Within specific soil substrate types, the effect of incubation temperature was most profound (*F* value ranges from 324.4 in WT soil to 689.15 in ST soil), with the microbial inoculum and an interaction between microbial inoculum and incubation temperature imposing highly significant effects (*p *<* *.001; Table 3). Within given incubation temperatures, the soil substrate had the most profound effect on the daily *R*_s_ (*F* value ranges from 148.6 at 5°C to 225.5 at 25°C), with the effects of microbial inoculum and an interaction between soil substrate and microbial inoculum being highly significant (*p *<* *.001; Table 3).

**Table 3:** Summary of repeated measures ANOVAs (measurement time as a covariate) for testing the effects of treatment factors under specific soil substrate and under a given temperature on C mineralization rate (*R*_s_) and cumulative C mineralization (*R*_cum_) during a 61‐day incubation

Soil substrate origin	Factors	Daily *R*_s_			*R*_cum_			Incubation temperature (°C)	Factors	Daily *R*_s_			*R*_cum_		
		*df*	*F*	*p*	*df*	*F*	*p*			*df*	*F*	*p*	*df*	*F*	*p*
CT	Microbial inoculum (Micr)	2	17.2	<.001	2	15.4	<.001	5	Soil substrate (Soil)	2	148.6	<.001	2	132.8	<.001
	Temperature (Temp)	2	417.0	<.001	2	36.6	<.001		Microbial inoculum (Micr)	2	59.4	<.001	2	36.9	<.001
	Micr × Temp	4	11.4	<.001	4	7.75	<.001		Soil × Micr	4	20.1	<.001	4	17.6	<.001
	Error	33			33				Error	31			40		
WT	Microbial inoculum (Micr)	2	38.3	<.001	2	26.6	<.001	15	Soil substrate (Soil)	2	151.0	<.001	2	66.6	<.001
	Temperature (Temp)	2	324.4	<.001	2	34.6	<.001		Microbial inoculum (Micr)	2	21.1	<.001	2	19.3	<.001
	Micr × Temp	4	52.2	<.001	4	21.2	<.001		Soil × Micr	4	33.4	<.001	4	25.1	<.001
	Error	33			33				Error	31			40		
ST	Microbial inoculum (Micr)	2	44.3	<.001	2	26.0	<.001	25	Soil substrate (Soil)	2	225.2	<.001	2	74.9	<.001
	Temperature (Temp)	2	689.2	<.001	2	113.6	<.001		Microbial inoculum (Micr)	2	17.4	<.001	2	7.02	.003
	Micr × Temp	4	24.3	<.001	4	13.3	<.001		Soil × Micr	4	32.5	<.001	4	19.2	<.001
	Error	27			27				Error	31			40		

CT, cool temperate forest; WT, warm temperate forest; ST, subtropical forest.

**Figure 2:**
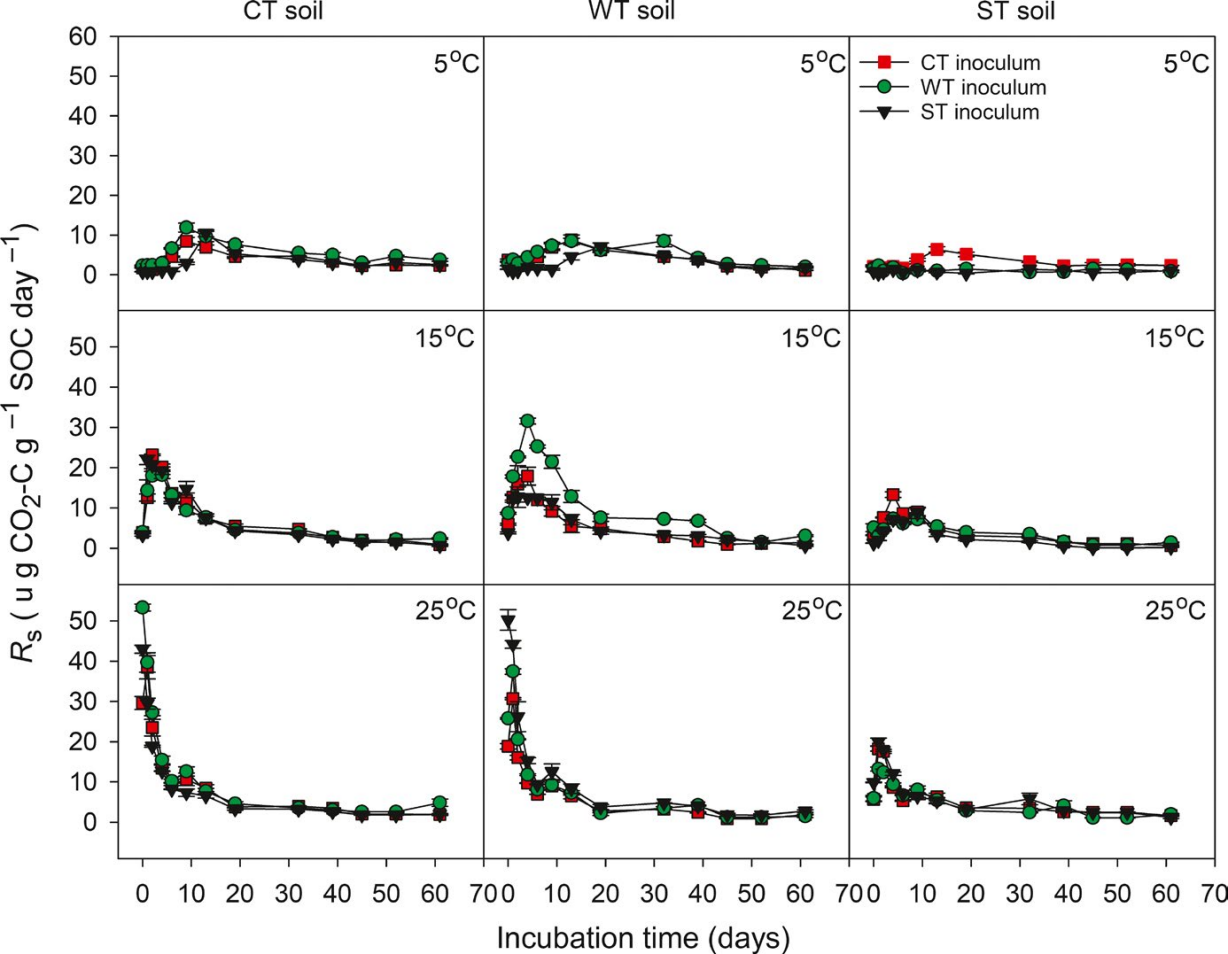
Changes in the rate of C mineralization during a 61‐day incubation period at three temperatures (5, 15, and 25°C) for combinations of soil substrates and microbial inocula from three climatically contrasting mixed‐wood forests. Values are normalized as per SOC. CT, cool temperate forest; WT, warm temperate forest; ST, subtropical forest. Vertical bars illustrate one standard error of means (*n* = 5)

**Figure 3:**
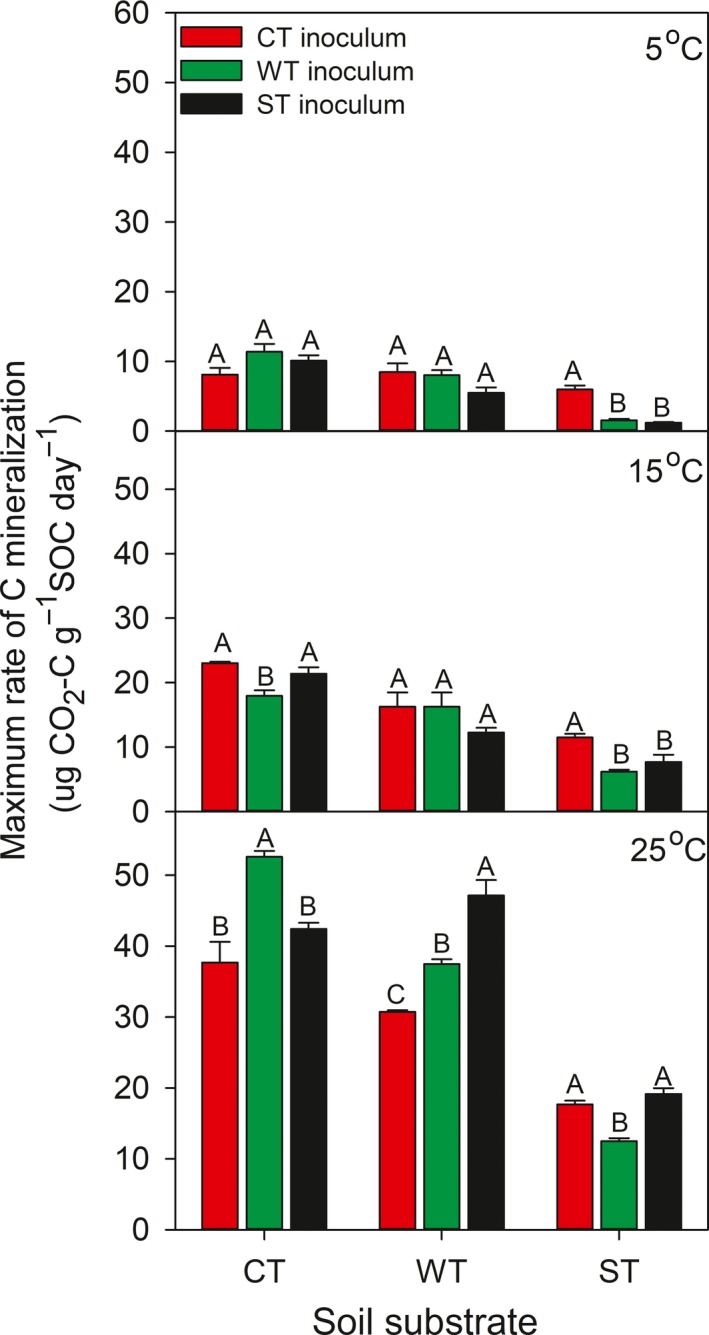
Maximum rate of carbon mineralization during a 61‐day incubation period at three temperatures (5, 15, and 25°C) for combinations of soil substrates and microbial inocula from three climatically contrasting mixed‐wood forests. Values are normalized as per SOC. CT, cool temperate forest; WT, warm temperate forest; ST, subtropical forest. Vertical bars illustrate one standard error of means (*n* = 5). Values designated with the same uppercase letters are not significantly different at *p *=* *.05

Over the 61‐day incubation period, the daily *R*_s_ varied with occurrence of a peak immediately or shortly after the commencement of inoculation and incubation, with the timing and magnitude of the peak differing among the three incubation temperatures and varied with soil substrate type and microbial inoculum treatment within given incubation temperatures (Figure 2). With decreases in the incubation temperature, there was generally a delay in the occurrence of the peak and a reduction in the maximum value of the daily *R*_s_. In given soil substrate, the average value of maximum *R*_s_ at 15 and 25°C was higher than that at 5°C (Figure 3). Among the three soil substrate types, the ST was lowest in the overall magnitude of daily *R*_s_ regardless of incubation temperature and microbial inoculum treatment. The microbial inoculum affected the maximum value of the daily rate of C mineralization within a given incubation temperature and soil substrate type (Figures 2 and 3). During the incubation period of 0–32 days, the incubation temperature had the greatest effects (*F *=* *1033.4) on the daily *R*_s_, followed by the soil substrate (*F *=* *432.8; Table 2); whereas during the incubation period 33–61 days, the soil substrate had the greatest effects (*F *=* *137.8), followed by the incubation temperature (*F *=* *59.9; Table 2).

**Figure 4:**
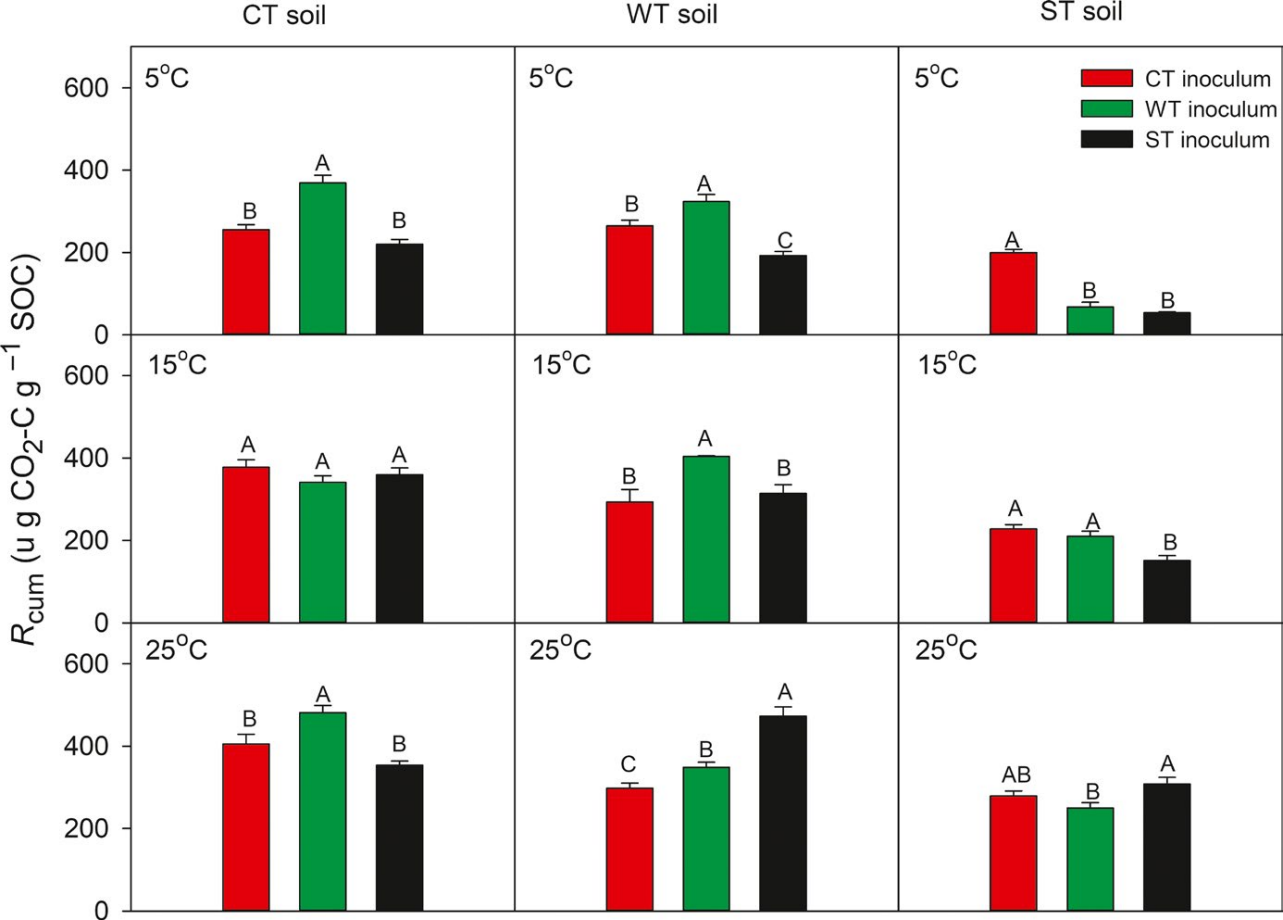
Cumulative carbon mineralization (*R*_cum_) over a 61‐day incubation period at three temperatures (5, 15, and 25°C) for combinations of soil substrate and microbial inocula originated from three climatically contrasting mixed‐wood forests. Values are normalized as per SOC. CT, cool temperate forest; WT, warm temperate forest; ST, subtropical forest. Vertical bars illustrate one standard error of means (*n* = 5). Values designated with the same uppercase letters are not significantly different at *p *=* *.05

*R*_cum_ was predominantly affected by the incubation temperature (*F* value ranges from 34.6 in WT soil to 113.6 in ST soil) within specific soil substrates, with the effects of microbial inoculum and an interaction between microbial inoculum and incubation temperature being equally secondary, albeit statistically highly significant (*p *<* *.001; Table 3). Under given incubation temperatures, the soil substrate had a predominant effect (*F* value ranges from 66.6 at 25°C to 132.8 at 5°C), with the relative effects of microbial inoculum and an interaction between soil substrate and microbial inoculum varying depending on incubation temperatures (Table 3). *R*_cum_ was consistently and significantly smaller (*p *<* *.05) in the ST soil substrate than in other two soil substrate types across the three incubation temperatures, and overall, was greatest at 25°C and smallest at 5°C, regardless of microbial inoculum treatment (Figure 4). In the CT substrate, significantly (*p *<* *.05) greater amount of SOC was mineralized by introduction of the WT inoculum than the other two inoculum types when incubated at either 25°C or 5°C, whereas there was no effect of the microbial inoculum at 15°C (Figure 4). In the WT substrate, the effect of microbial inoculum varied with incubation temperatures; at 25°C, the ST inoculum resulted in greatest cumulative C mineralization, followed by the WT inoculum; at 15°C, the WT inoculum resulted in significantly (*p *<* *.05) greater *R*_cum_ than the other two inoculum types; while at the 5°C, the WT resulted greatest *R*_cum_, followed by the CT inoculum (Figure 3). In the ST substrate, the effect on *R*_cum_ ranked in the order of the ST inoculum > the CT inoculum > the WT inoculum but without much variation at 25°C; at 15°C, both the CT and the WT inocula resulted in significantly (*p *<* *.05) greater *R*_cum_ than the ST inoculum; while at 5°C, the CT inoculum induced significantly (*p *<* *.05) and markedly greater *R*_cum_ than the other two microbial inocula (Figure 4).

### 3.3 Changes in microbial community structure and soil enzyme activities

**Figure 5:**
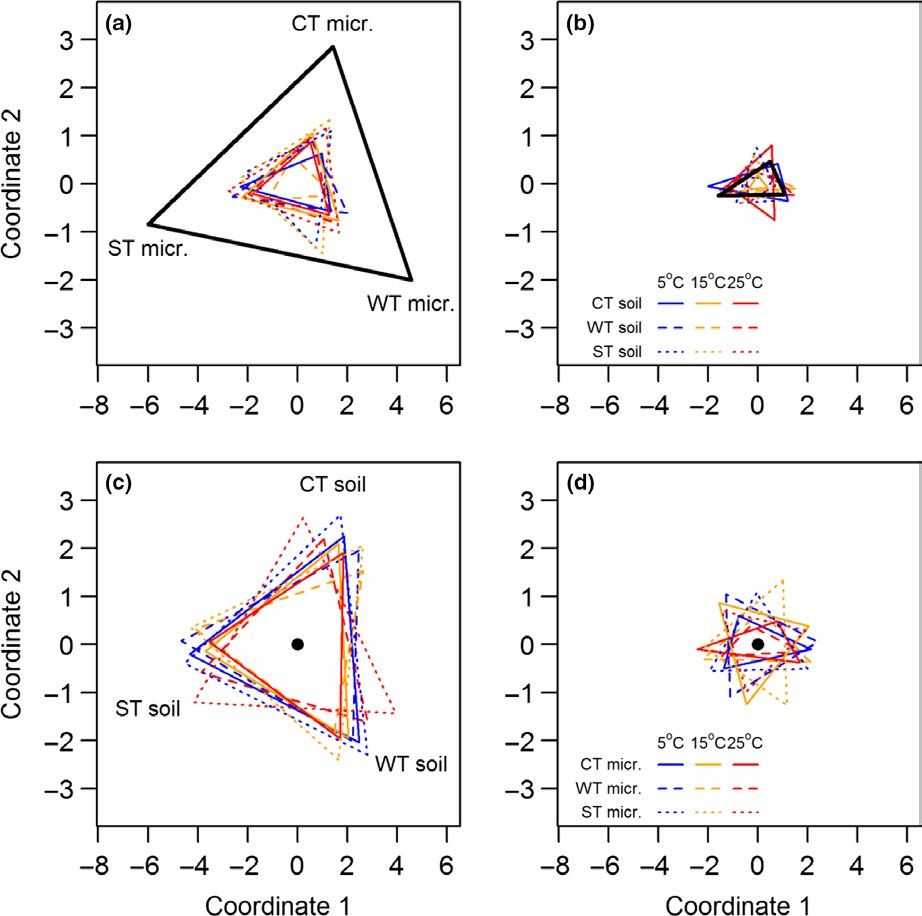
Changes in the dissimilarity of microbial community structure (a) and soil enzyme activity (b) when different microbial inocula were introduced to a specific soil substrate, and changes in the dissimilarity of microbial community structure (c) and soil enzyme activity (d) when a specific microbial inoculums was introduced to different soil substrates, over the incubation period at three temperatures (5, 15, and 25°C). The bold black open triangles in panels (a) and (b) show the dissimilarity among the original soil samples, and the bold black dots in panels (c) and (d) show a lack of active microbial community prior to microbial inoculation

**Figure 6:**
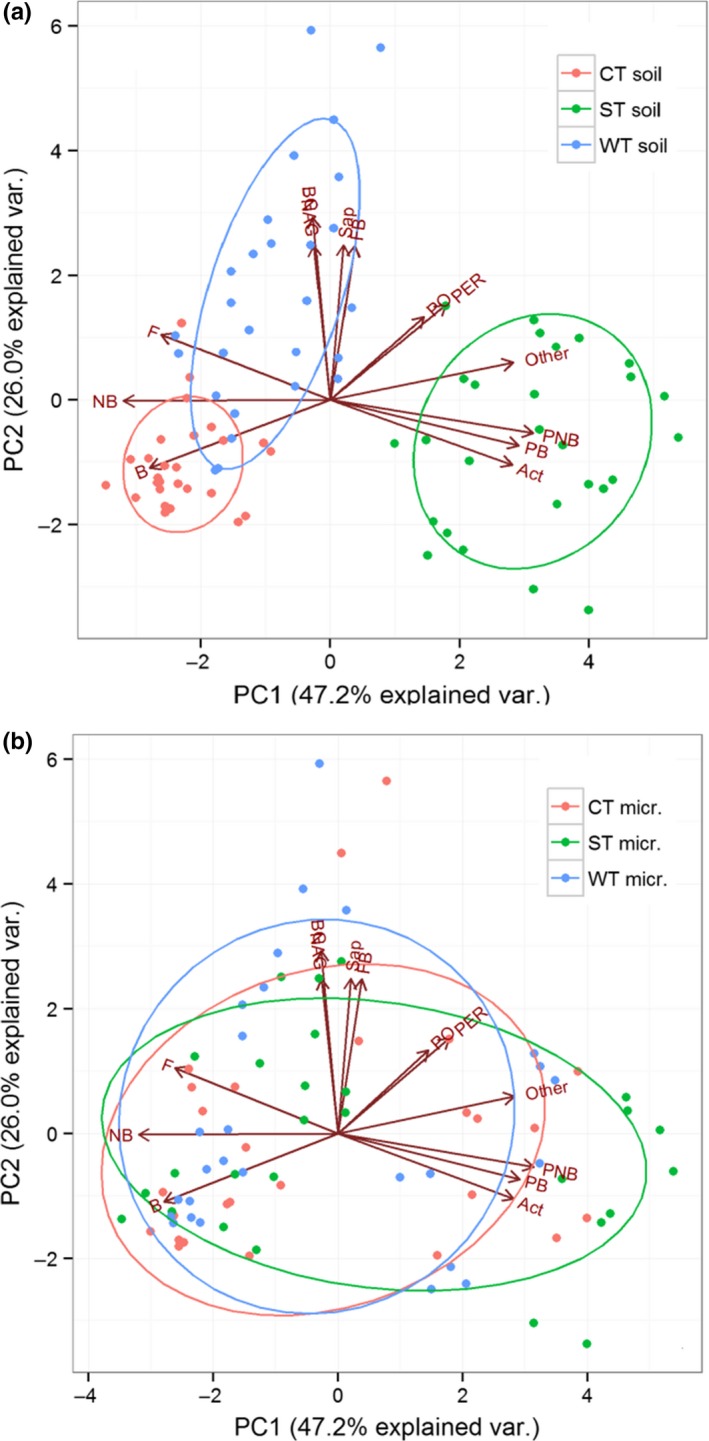
Ordination of Principal Component Analysis (PCA) showing 13 microbial variables (community structure and enzyme activities) in relation to different soil substrates: (a) and different microbial inocula (b) at the end of incubation period. *B*, total bacteria; *PB*, gram‐positive bacteria group; *NB*, gram‐negative bacteria group; *PNB*, ratio of gram‐positive bacteria group to gram‐negative group; *Act*, actinomycete; *Sap*, saprophytic fungi; *F*, arbuscular mycorrhizal fungi; *FB*, ratio of fungi to bacteria ratio; *other*, unidentified microbial group; *PO*, phenol oxidase; *PER*, peroxidase; *BG*, β‐1,4‐glucosidase; *NAG*,* N*‐acetyl‐β‐glucosaminidase

There was convergence of microbial community structure (Figure 5a) with unchanged soil enzyme activity when three different sources of microbial inocula were introduced to a specific type of soil substrate (Figure 5b). The original microbial community structures of three soils show clear dissimilarities, as illustrated by the size of the bold black triangle in Figure 6a, but the dissimilarities were greatly reduced following a 61‐day incubation for various combinations of soil substrates and incubation temperatures when different microbial inocula were introduced to a specific soil substrate (Figure 5a), while no such differentiations and changes were found for soil enzyme activities of the same treatments (Figure 5b). In contrast, both the microbial community structure and soil enzyme activity diverged when a specific source of microbial inoculum was introduced to three different soil substrates, as illustrated by changes in the bold black dots into enlarged triangles in Figure 5c,d.

The results of PCA on soil samples at the end of the incubation period show clear separation of microbial community structure and soil enzyme activities among the three soils (Figure 6a), but not among the three microbial inocula (Figure 6b); the axes 1 and 2 explain 47.2% and 26.0% of total variation, respectively (Figure 6). The CT soil is typically associated with the total soil bacteria and the gram‐negative bacteria group; the ST soil is more linked to the actinomycete, gram‐positive bacteria group and the ratio of gram‐positive bacteria group to gram‐negative bacteria group; the WT soil is strongly associated with the saprophytic fungi, arbuscular mycorrhizal fungi, and the fungi to bacteria ratio (Figure 6a). The hydrolytic enzyme activities (BG and NAG) are more closely related to the WT soil, whereas the oxidative enzyme activities (PO and PER) are more closely related to the ST soil (Figure 6a). No clear pattern was observed when the same microbial inocula were incorporated into different soils (Figure 6b).

## 4 DISCUSSION

The daily rate and cumulative quantity of C mineralization were strongly affected by incubation temperature, soil substrate, source of microbial inoculum, and their interactions over a 61‐day incubation period in laboratory. Among the treatment factors, temperature had the strongest effect on the temporal dynamics of soil C mineralization; a decrease in the incubation temperature from 25 to 5°C resulted in a delayed peak and reduced magnitude of the maximum rate of C mineralization during the study period. This is consistent with previous findings that climate exerts dominant controls on SOM decomposition (Carvalhais et al., 2014; Kirschbaum, 2004; Sun et al., 2004). A recent synthesis by Luo, Feng, Luo, Baldock, and Wang (2017) showed that climate (precipitation and temperature) accounted for as much as 25% of the relative influence on SOC by various environmental, soil biotic, and abiotic factors. Within given temperatures, however, we found that soil substrate had much greater influence on the rate of soil C mineralization than microbial inoculum, suggesting the importance of soil quality in determining the soil C mineralization—possibly the microbial adaptation to the soil matrix. The strong effect of temperature on the rate of soil C mineralization does not rule out the importance of soil microbial community, as it is recognized that climate and environmental factors can mask the influence of decomposer community on decomposition, due to the fact that soil microorganisms may both adapt to and be affected by climate and environments (Canarini, Carrillo, Mariotte, Ingram, & Dijkstra, 2016; Keiser & Bradford, 2017). Moreover, the structure and functions of soil microbial communities are further constrained by soil physiochemical properties and SOM quality (Fabian, Zlatanovic, Mutz, & Premke, 2017; Sun et al., 2016; Xun et al., 2015; You et al., 2014, 2016). Growing evidences show that soil geochemistry and physical structure impose direct effects on SOM stability by creating physiochemical barriers preventing microorganisms to access carbon sources (Bardgett et al., 2008; Chenu & Plante, 2006; Delgado‐Baquerizo et al., 2015; Doetterl et al., 2015; Plante, Conant, Stewart, Paustian, & Six, 2006). For SOM decomposition in mineral soils, it has been suggested that microbial community structure “is likely not important” because soil physical protection is more important than microbial community (Schimel & Schaeffer, 2012).

The temporal dynamics of soil C mineralization was characterized by occurrence of a peak in the daily rate of C mineralization following the commencement of the inoculation and incubation; the marked peaks and fluctuation of daily rate of soil C mineralization were similar to the findings of other incubation experiments, including cases when sterilized soil was mixed with nonsterilized soil (Fan, Huang, Tang, Li, & Liang, 2012; Nie et al., 2013) or when sterilized litter was mixed with soil (Fanin & Bertrand, 2016; Strickland et al., 2009). Similar phenomena have been found in incubation experiments with untreated field soils (Ci, Al‐Kaisi, Wang, Ding, & Xie, 2015; Zhou et al., 2013). So, the change in soil carbon mineralization in the initial phase after inoculating maybe complex and affected by many factors but not a specific result of our study. The occurrence of the peak may be a combined result of the likely biophysical degradation of the labile soil organic C by autoclaving (Nie et al., 2013) and microbial colonization of the sterilized soil substrate. In this study, a decrease in the incubation temperature from 25 to 5°C resulted in a delayed peak and reduced magnitude of the maximum rate of C mineralization during the incubation period, and the timing of peaks and maximum value of daily rate of soil C mineralization also varied with soil substrate. Similar findings have been reported in the literature (e.g., Bradford et al., 2008; Shaver et al., 2006; Wetterstedt, Persson, & Ågren, 2010; Zhou et al., 2013).

We divided the 61‐day incubation into two periods, that is, the initial period of microbial colonization and active C mineralization (Day 0–32) and the period of settled microbial community and constrained C mineralization (Day 33–61), and found different effects of treatment factors on the daily rate of C mineralization. The former period was predominantly affected by the incubation temperature, and the latter by the soil substrate. The predominant temperature control of C mineralization during the early laboratory incubation may be explained by the ability of microbial communities to colonize sterilized soils (Bradford et al., 2008; Rustad et al., 2001), hence simpling an acclimation of microbial‐driven C mineralization (Luo, Wan, Hui, & Wallace, 2001). With further progressing of the incubation, substrate supply limitation outweighs the environmental constraints on C mineralization (Luo et al., 2017; Wang, Dalal, Moody, & Smith, 2003).

The cumulative soil C mineralization during our experimental period was differently affected by the treatment factors compared to the daily rate of soil C mineralization, such that the soil substrate prevailed as the most influential factor, with the incubation temperature being secondary and microbial inoculum being the weakest. During the experiment, the incubation treatment lasted for 61 days and the daily rate of soil C mineralization nearly diminished toward the end of the experiment. Therefore, the cumulative C mineralization in our study reflected the mineralizable labile SOC under given conditions. There are studies demonstrating that soil physiochemical properties are the primary determinant of potential C mineralization, but the realizable C mineralization is strongly dependent on both the decomposer community and the environmental conditions that shape the decomposer community and affect the soil microbial function (e.g., Canarini et al., 2016; Fabian et al., 2017; Keiser & Bradford, 2017; Xun et al., 2015; You et al., 2014).

In this study, when different sources of microbial inocula were introduced to the same soil substrate, the microbial community structure converged following incubation without much affecting the soil enzyme activities, whereas when different types of soil substrate were inoculated with the same sources of microbial inocula, both the microbial community structure and soil enzyme activities diverged. Our findings demonstrate that soil microbial community structure is strongly shaped by soil physiochemical properties, and soil C mineralization is constrained by both soil physiochemical properties and soil microbial community. The significant effects of soil substrate, microbial inoculum, incubation temperature, and their various interactions on the daily rate and cumulative amount of C mineralization highlighted the complex controls of biotic and abiotic factors on soil C transformation and turnover.

Our results also show that the effects of soil substrate on microbial community structure are likely a result of constraints by interactions between physiochemical properties and biotic factors (Burke, Weintraub, Hewins, & Kalisz, 2011). For example, the cool temperate forest soils with rich SOM and better development were closely associated with the total bacteria and gram‐negative bacteria group, similar to the findings of other studies (Balser & Firestone, 2005; Kramer & Gleixner, 2008; You et al., 2014). The subtropical soils, being more acidic, were strongly associated with actino‐bacteria—a metabolically versatile group of microorganisms that degrade lignin and cellulose (Rousk et al., 2010). Our previous studies well established that biotic and environmental factors control soil C transformation and turnover by shaping the soil microbial structure (Sun et al., 2016; You et al., 2014, 2016).

While the climatic controls and effects of vegetation on soil microbial structure and function are widely studied (e.g., Brockett et al., 2012; Hackl et al., 2005; You et al., 2016), the interactive effects of climate and local factors in shaping the soil microbial community have received far less recognition. Geographical separations, soil physiochemical properties, and prevailing environmental factors seem all play important roles in constraining the microbial adaption to the soil matrix. Our findings show that the microbial decomposition of SOC is generally more enhanced by matching incubation temperature to the prevailing condition of soil substrate and microbial inoculum origins, but when the reciprocal inoculations were made between soils with greater geographical separation and greater differences in soil physiochemical properties, the effects appear to be none or negative. Future researches are required to address the interactive effects of geographical separation, climate, vegetation, and soil pedology on soil microbial structure and function in order to understand the responses and adaptation of soils to global change.

It needs to be pointed out that, due to lack of strict controls on the quantity of microbial community used for inoculation, some of the variations in temperature responses of soil C decomposition and cross‐soil differences may partially reflect natural variations and recolonization capacity of soil microbial communities among forest sites. Therefore, some of our results require verification by better controlled experimental approaches. Nonetheless, our findings provide new evidence of the relative importance of soil substrate and microbial community and interaction with temperature in affecting soil C mineralization, microbial community structure, and soil enzyme activities. Overall, temperature plays a predominant role in affecting the rate of soil C mineralization, while soil substrate determines the mineralizable SOC under given conditions. The role of microbial community in driving SOC mineralization is only secondary in comparison with climate and soil substrate, as soil microbial community is both affected, and adapts to, climatic factors and soil matrix. However, the quantitative contributions are still relatively unclear. Research efforts are needed for improved methodology and adoption of new technology such as ^13^C labeling technique and new autoclave, etc. Uncertainty in the effectiveness of autoclaving and microbial recolonization of reciprocally inoculated soils remain to be better elucidated.

## CONFLICT OF INTEREST

None declared.

## AUTHOR CONTRIBUTIONS

ZT designed and performed the experiment. ZT and XS collected soil samples and conducted laboratory analysis. ZL performed data analysis. NH provided laboratory devices and advised on experimental procedures. ZT, ZL, NH and OJS wrote the manuscript.

## References

[ece33708-bib-0001] Baldrian, P. (2009). Microbial enzyme‐catalyzed processes in soils and their analysis. Plant, Soil and Environment, 55, 370–378.

[ece33708-bib-0002] Balser, T. C. , & Firestone, M. K. (2005). Linking microbial community composition and soil processes in a California annual grassland and mixed‐conifer forest. Biogeochemistry, 73, 395–415. https://doi.org/10.1007/s10533-004-0372-y

[ece33708-bib-0003] Bardgett, R. D. , Freeman, C. , & Ostle, N. J. (2008). Microbial contributions to climate change through carbon cycle feedbacks. The ISME Journal, 2, 805–814. https://doi.org/10.1038/ismej.2008.58 18615117 10.1038/ismej.2008.58

[ece33708-bib-0004] Bardgett, R. D. , & van der Putten, W. H. (2014). Belowground biodiversity and ecosystem functioning. Nature, 515, 505–511. https://doi.org/10.1038/nature13855 25428498 10.1038/nature13855

[ece33708-bib-0005] Bossio, D. A. , & Scow, K. M. (1998). Impacts of carbon and flooding on soil microbial communities: Phospholipid fatty acid profiles and substrate utilization patterns. Microbial Ecology, 35, 265–278. https://doi.org/10.1007/s002489900082 9569284 10.1007/s002489900082

[ece33708-bib-0006] Bradford, M. A. , Davies, C. A. , Frey, S. D. , Maddox, T. R. , Melillo, J. M. , Mohan, J. E. , … Wallenstein, M. D. (2008). Thermal adaptation of soil microbial respiration to elevated temperature. Ecology Letters, 11, 1316–1327. https://doi.org/10.1111/j.1461-0248.2008.01251.x 19046360 10.1111/j.1461-0248.2008.01251.x

[ece33708-bib-0007] Brockett, B. F. T. , Prescott, C. E. , & Grayston, S. J. (2012). Soil moisture is the major factor influencing microbial community structure and enzyme activities across seven biogeoclimatic zones in western Canada. Soil Biology and Biochemistry, 44, 9–20. https://doi.org/10.1016/j.soilbio.2011.09.003

[ece33708-bib-0008] Burke, D. J. , Weintraub, M. N. , Hewins, C. R. , & Kalisz, S. (2011). Relationship between soil enzyme activities, nutrient cycling and soil fungal communities in a northern hardwood forest. Soil Biology and Biochemistry, 43, 795–803. https://doi.org/10.1016/j.soilbio.2010.12.014

[ece33708-bib-0009] Canarini, A. , Carrillo, Y. , Mariotte, P. , Ingram, L. , & Dijkstra, F. A. (2016). Soil microbial community resistance to drought and links to C stabilization in an Australian grassland. Soil Biology and Biochemistry, 103, 171–180. https://doi.org/10.1016/j.soilbio.2016.08.024

[ece33708-bib-0010] Carvalhais, N. , Forkel, M. , Khomik, M. , Bellarby, J. , Jung, M. , Migliavacca, M. , … Reichstein, M. (2014). Global covariation of carbon turnover times with climate in terrestrial ecosystems. Nature, 514, 213–217. 25252980 10.1038/nature13731

[ece33708-bib-0011] Chenu, C. , & Plante, A. F. (2006). Clay‐sized organo‐mineral complexes in a cultivation chronosequence: Revisiting the concept of the ‘primary organo‐mineral complex’. European Journal of Soil Science, 57, 596–607. https://doi.org/10.1111/j.1365-2389.2006.00834.x

[ece33708-bib-0012] Ci, E. , Al‐Kaisi, M. M. , Wang, L. G. , Ding, C. H. , & Xie, D. T. (2015). Soil organic carbon mineralization as affected by cyclical temperature fluctuations in a karst region of southwestern China. Pedosphere, 25, 512–523. https://doi.org/10.1016/S1002-0160(15)30032-1

[ece33708-bib-0013] Cong, J. , Yang, Y. F. , Liu, X. D. , Lu, H. , Liu, X. , Zhou, J. Z. , … Zhang, Y. G. (2015). Analyses of soil microbial community compositions and functional genes reveal potential consequences of natural forest succession. Scientific Reports, 5, 10007. 25943705 10.1038/srep10007 PMC4421864

[ece33708-bib-0501] Delgado‐Baquerizo, M. , García‐Palacios, P. , Milla, R. , Gallardo, A. , & Maestre, F. T. (2015). Soil characteristics determine soil carbon and nitrogen availability during leaf litter decomposition regardless of litter quality. Soil Biology and Biochemistry, 81, 134–142. https://doi.org/10.1016/j.soilbio.2014.11.009

[ece33708-bib-0014] Drenovsky, R. E. , Steenwerth, K. L. , Jackson, L. E. , & Scow, K. M. (2010). Land use and climatic factors structure regional patterns in soil microbial communities. Global Ecology and Biogeography, 19, 27–39. https://doi.org/10.1111/j.1466-8238.2009.00486.x 24443643 10.1111/j.1466-8238.2009.00486.x PMC3891896

[ece33708-bib-0502] Doetterl, S. , Stevens, A. , Six, J. , Merckx, R. , Van Oost, K. , Pinto, M. C. , … Boeckx, P. (2015). Soil carbon storage controlled by interactions between geochemistry and climate. Nature Geoscience, 8, 780–783.

[ece33708-bib-0015] Fabian, J. , Zlatanovic, S. , Mutz, M. , & Premke, K. (2017). Fungal‐bacterial dynamics and their contribution to terrigenous carbon turnover in relation to organic matter quality. The ISME Journal, 11, 415–425. https://doi.org/10.1038/ismej.2016.131 27983721 10.1038/ismej.2016.131 PMC5270572

[ece33708-bib-0016] Fan, F. L. , Huang, P. R. , Tang, Y. J. , Li, Z. J. , & Liang, Y. C. (2012). Altered microbial communities change soil respiration rate and their temperature sensitivity. Environmental Sciences, 33, 932–937. (in Chinese with English abstract). 22624390

[ece33708-bib-0017] Fanin, N. , & Bertrand, I. (2016). Aboveground litter quality is a better predictor than belowground microbial communities when estimating carbon mineralization along a land‐use gradient. Soil Biology and Biochemistry, 94, 48–60. https://doi.org/10.1016/j.soilbio.2015.11.007

[ece33708-bib-0018] Foesel, B. U. , Nägele, V. , Naether, A. , Wüst, P. K. , Weinert, J. , Bonkowski, M. , … Overmann, J. (2014). Determinants of Acidobacteria activity inferred from the relative abundances of 16S rRNA transcripts in German grassland and forest soils. Environmental Microbiology, 16, 658–675. https://doi.org/10.1111/1462-2920.12162 23802854 10.1111/1462-2920.12162

[ece33708-bib-0019] Gallaher, R. N. , Weldon, C. O. , & Boswell, F. C. (1976). A semiautomated procedure for total nitrogen in plant and soil samples. Soil Science Society of America Journal, 40, 887–889. https://doi.org/10.2136/sssaj1976.03615995004000060026x

[ece33708-bib-0020] Gee, G. W. , Bauder, J. W. , & Klute, A. (1986). Particle‐size analysis In Klute A. (Ed.), Methods of soil analysis, Part 1. Physical and mineralogical methods. Agronomy Monograph No. 9, 2nd ed. (pp. 383–411). Madison, WI: American Society of Agronomy and Soil Science Society of America.

[ece33708-bib-0021] Hackl, E. , Pfeffer, M. , Donat, C. , Bachmann, G. , & Zechmeister‐Boltenstern, S. (2005). Composition of the microbial communities in the mineral soil under different types of natural forest. Soil Biology and Biochemistry, 37, 661–671. https://doi.org/10.1016/j.soilbio.2004.08.023

[ece33708-bib-0022] He, N. P. , Wang, R. M. , Gao, Y. , Dai, J. Z. , Wen, X. F. , & Yu, G. R. (2013). Changes in the temperature sensitivity of SOM decomposition with grassland succession: Implications for soil C sequestration. Ecology and Evolution, 3, 5045–5054. 24455135 10.1002/ece3.881 PMC3892367

[ece33708-bib-0023] van der Heijden, M. G. A. , Bardgett, R. D. , & Van Straalen, N. M. (2008). The unseen majority: Soil microbes as drivers of plant diversity and productivity in terrestrial ecosystems. Ecology Letters, 11, 296–310. https://doi.org/10.1111/j.1461-0248.2007.01139.x 18047587 10.1111/j.1461-0248.2007.01139.x

[ece33708-bib-0024] Keiluweit, M. , Bougoure, J. J. , Nico, P. S. , Pett‐Ridge, J. , Weber, P. K. , & Kleber, M. (2015). Mineral protection of soil carbon counteracted by root exudates. Nature Climate Change, 5, 588–595. https://doi.org/10.1038/nclimate2580

[ece33708-bib-0025] Keiser, A. D. , & Bradford, M. A. (2017). Climate masks decomposer influence in a cross‐site litter decomposition study. Soil Biology and Biochemistry, 107, 180–187. https://doi.org/10.1016/j.soilbio.2016.12.022

[ece33708-bib-0026] Kirschbaum, M. U. F. (2004). Soil respiration under prolonged soil warming: Are rate reductions caused by acclimation or substrate loss? Global Change Biology, 10, 1870–1877. https://doi.org/10.1111/j.1365-2486.2004.00852.x

[ece33708-bib-0027] Kramer, C. , & Gleixner, G. (2008). Soil organic matter in soil depth profiles: Distinct carbon preferences of microbial groups during carbon transformation. Soil Biology and Biochemistry, 40, 425–433. https://doi.org/10.1016/j.soilbio.2007.09.016

[ece33708-bib-0028] Kruskal, J. B. (1964). Nonmetric multidimensional scaling: A numerical method. Psychometrika, 29, 115–129. https://doi.org/10.1007/BF02289694

[ece33708-bib-0029] Li, X. D. , Coles, B. J. , Ramsey, M. H. , & Thornton, I. (1995). Sequential extraction of soils for multielement analysis by ICP‐AES. Chemical Geology, 124, 109–123. https://doi.org/10.1016/0009-2541(95)00029-L

[ece33708-bib-0030] Li, J. , He, N. , Xu, L. , Chai, H. , Liu, Y. , Wang, D. , … Sun, X. (2017). Asymmetric responses of soil heterotrophic respiration to rising and decreasing temperatures. Soil Biology and Biochemistry, 106, 18–27.

[ece33708-bib-0503] Li, X. F. , Han, S. J. , Guo, Z. L. , Shao, D. K. , & Xin, L. H. (2010). Changes in soil microbial biomass carbon and enzyme activities under elevated CO2 affect fine root decomposition processes in a Mongolian oak ecosystem. Soil Biology and Biochemistry, 42, 1101–1107.

[ece33708-bib-0031] Liu, Y. , He, N. P. , Zhu, J. X. , Xu, L. , Yu, G. R. , Niu, S. L. , … Wen, X. F. (2016). Regional variation in the temperature sensitivity of soil organic matter decomposition in China's forests and grasslands. Global Change Biology, 23, 3393–3402. 10.1111/gcb.13613 28055123

[ece33708-bib-0032] Luo, Z. K. , Feng, W. T. , Luo, Y. Q. , Baldock, J. , & Wang, E. L. (2017). Soil organic carbon dynamics jointly controlled by climate, carbon inputs, soil properties and soil carbon fractions. Global Change Biology, 23, 4430–4439. https://doi.org/10.1111/gcb.13767 28544252 10.1111/gcb.13767

[ece33708-bib-0033] Luo, Y. Q. , Wan, S. Q. , Hui, D. F. , & Wallace, L. L. (2001). Acclimatization of soil respiration to warming in a tall grass prairie. Nature, 413, 622–625. https://doi.org/10.1038/35098065 11675783 10.1038/35098065

[ece33708-bib-0034] McGuire, K. L. , & Treseder, K. K. (2010). Microbial communities and their relevance for ecosystem models: Decomposition as a case study. Soil Biology and Biochemistry, 42, 529–535. https://doi.org/10.1016/j.soilbio.2009.11.016

[ece33708-bib-0035] Nelson, D. W. , & Sommers, L. E. (1982). Total carbon, organic carbon, and organic matter In Page A. L., Miller R. H., & Keeney D. R. (Eds.), Methods of soil analysis (pp. 101–129). Madison: American Society of Agronomy and Soil Science Society of American.

[ece33708-bib-0036] Nie, M. , Pendall, E. , Bell, C. , Gasch, C. K. , Raut, S. , Tamang, S. , & Wallenstein, M. D. (2013). Positive climate feedbacks of soil microbial communities in a semi‐arid grassland. Ecology Letters, 16, 234–241. https://doi.org/10.1111/ele.12034 23157642 10.1111/ele.12034

[ece33708-bib-0037] Parham, J. A. , & Deng, S. P. (2000). Detection, quantification and characterization of β‐glucosaminidase activity in soil. Soil Biology and Biochemistry, 32, 1183–1190. https://doi.org/10.1016/S0038-0717(00)00034-1

[ece33708-bib-0038] Plante, A. F. , Conant, R. T. , Stewart, C. E. , Paustian, K. , & Six, J. (2006). Impact of soil texture on the distribution of soil organic matter in physical and chemical fractions. Soil Science Society of America Journal, 70, 287–296. https://doi.org/10.2136/sssaj2004.0363

[ece33708-bib-0039] Prescott, C. E. , & Grayston, S. J. (2013). Tree species influence on microbial communities in litter and soil: Current knowledge and research needs. Forest Ecology and Management, 309, 19–27. https://doi.org/10.1016/j.foreco.2013.02.034

[ece33708-bib-0040] Reinsch, S. , Michelsen, A. , Sárossy, Z. , Egsgaard, H. , Kappel Schmidt, I. , Jakobsen, I. , & Ambus, P. (2013). Will anticipated future climatic conditions affect belowground C utilization?—Insights into the role of microbial functional groups in a temperate heath/grassland. Cell, 149, 467–482.

[ece33708-bib-0041] Rousk, J. , Baath, E. , Brookes, P. C. , Lauber, C. L. , Lozupone, C. , Caporaso, J. G. , … Fierer, N. (2010). Soil bacterial and fungal communities across a pH gradient in an arable soil. The ISME Journal, 4, 1340–1351. https://doi.org/10.1038/ismej.2010.58 20445636 10.1038/ismej.2010.58

[ece33708-bib-0042] Rustad, L. E. , Campbell, J. L. , Marion, G. M. , Norby, R. J. , Mitchell, M. J. , Hartley, A. E. , … GTCE‐NEWS . (2001). A meta‐analysis of the response of soil respiration, net nitrogen mineralization, and aboveground plant growth to experimental ecosystem warming. Oecologia, 126, 543–562. https://doi.org/10.1007/s004420000544 28547240 10.1007/s004420000544

[ece33708-bib-0043] Schimel, J. P. , & Schaeffer, S. M. (2012). Microbial control over carbon cycling in soil. Frontiers in Microbiology, 3, 348. 23055998 10.3389/fmicb.2012.00348 PMC3458434

[ece33708-bib-0044] Schmidt, M. W. , Torn, M. S. , Abiven, S. , Dittmar, T. , Guggenberger, G. , Janssens, I. A. , … Trumbore, S. E. (2011). Persistence of soil organic matter as an ecosystem property. Nature, 478, 49–56. https://doi.org/10.1038/nature10386 21979045 10.1038/nature10386

[ece33708-bib-0045] Shaver, G. R. , Giblin, A. E. , Nadenlhoffer, K. J. , Thieler, K. K. , Downs, M. R. , Launder, J. A. , & Rastetter, E. B. (2006). Carbon turnover in Alaskan tundra soils: Effects of organic matter quality, temperature, moisture and fertilizer. Journal of Ecology, 94, 740–753. https://doi.org/10.1111/j.1365-2745.2006.01139.x

[ece33708-bib-0046] Sinsabaugh, R. L. , Antibus, R. K. , Linkins, A. E. , McClaugherty, C. A. , Rayburn, L. , Repert, D. , & Weiland, T. (1993). Wood decomposition: Nitrogen and phosphorus dynamics in relation to extracellular enzyme activity. Ecology, 74, 1586–1593. https://doi.org/10.2307/1940086

[ece33708-bib-0047] Strickland, M. S. , Lauber, C. , Fierer, N. , & Bradford, M. A. (2009). Testing the functional significance of microbial community composition. Ecology, 90, 441–451. https://doi.org/10.1890/08-0296.1 19323228 10.1890/08-0296.1

[ece33708-bib-0048] Sun, O. J. , Campbell, J. , Law, B. E. , & Wolf, V. (2004). Dynamics of carbon stocks in soils and detritus across chronosequences of different forest types in the Pacific Northwest, USA. Global Change Biology, 10, 1470–1481. https://doi.org/10.1111/j.1365-2486.2004.00829.x

[ece33708-bib-0049] Sun, X. L. , Zhao, J. , You, Y. M. , & Sun, O. J. (2016). Soil microbial responses to forest floor litter manipulation and nitrogen addition in a mixed‐wood forest of northern China. Scientific Reports, 6, 19536 https://doi.org/10.1038/srep19536 26762490 10.1038/srep19536 PMC4725858

[ece33708-bib-0050] Toriyama, J. , Hak, M. , Imaya, A. , Hirai, K. , & Kiyono, Y. (2015). Effects of forest type and environmental factors on the soil organic carbon pool and its density fractions in a seasonally dry tropical forest. Forest Ecology and Management, 335, 147–155. https://doi.org/10.1016/j.foreco.2014.09.037

[ece33708-bib-0051] Vance, E. D. , Brookes, P. C. , & Jenkinson, D. S. (1987). An extraction method for measuring soil microbial biomass C. Soil Biology and Biochemistry, 19, 703–707. https://doi.org/10.1016/0038-0717(87)90052-6

[ece33708-bib-0052] van de Voorde, T. F. J. , van der Putten, W. H. , & Bezemer, T. M. (2012). Soil inoculation method determines the strength of plant–soil interactions. Soil Biology and Biochemistry, 55, 1–6. https://doi.org/10.1016/j.soilbio.2012.05.020

[ece33708-bib-0053] Waldrop, M. P. , & Firestone, M. K. (2004). Microbial community utilization of recalcitrant and simple carbon compounds: Impact of oak‐woodland plant communities. Oecologia, 138, 275–284. https://doi.org/10.1007/s00442-003-1419-9 14614618 10.1007/s00442-003-1419-9

[ece33708-bib-0054] Wan, X. H. , Huang, Z. Q. , He, Z. M. , Yu, Z. P. , Wang, M. H. , Davis, M. R. , & Yang, Y. S. (2015). Soil C: N ratio is the major determinant of soil microbial community structure in subtropical coniferous and broadleaf forest plantations. Plant and Soil, 387, 103–116. https://doi.org/10.1007/s11104-014-2277-4

[ece33708-bib-0055] Wang, W. J. , Dalal, R. C. , Moody, P. W. , & Smith, C. J. (2003). Relationships of soil respiration to microbial biomass, substrate availability and clay content. Soil Biology and Biochemistry, 35, 273–284. https://doi.org/10.1016/S0038-0717(02)00274-2

[ece33708-bib-0056] Wang, Q. , He, N. P. , Yu, G. R. , Gao, Y. , Wen, X. F. , Wang, R. F. , … Yu, Q. (2016). Soil microbial respiration rate and temperature sensitivity along a north‐south forest transect in eastern China: Patterns and influencing factors. Journal of Geophysical Research‐Biogeosciences, 121, 399–410. https://doi.org/10.1002/2015JG003217

[ece33708-bib-0057] Wetterstedt, J. Å. M. , Persson, T. , & Ågren, G. I. (2010). Temperature sensitivity and substrate quality in soil organic matter decomposition: Results of an incubation study with three substrates. Global Change Biology, 16, 1806–1819.

[ece33708-bib-0058] Xu, X. B. , Schimel, J. P. , Thornton, P. E. , Song, X. , Yuan, F. M. , & Goswami, S. (2014). Substrate and environmental controls on microbial assimilation of soil organic carbon: A framework for Earth system models. Ecology Letters, 17, 547–555. https://doi.org/10.1111/ele.12254 24529215 10.1111/ele.12254

[ece33708-bib-0059] Xun, W. B. , Huang, T. , Zhao, J. , Ran, W. , Wang, B. R. , Shen, Q. R. , & Zhang, R. F. (2015). Environmental conditions rather than microbial inoculum composition determine the bacterial composition, microbial biomass and enzymatic activity of reconstructed soil microbial communities. Soil Biology and Biochemistry, 90, 10–18. https://doi.org/10.1016/j.soilbio.2015.07.018

[ece33708-bib-0060] You, Y. M. , Wang, J. , Huang, X. M. , Tang, Z. X. , Liu, S. R. , & Sun, O. J. (2014). Relating microbial community structure to functioning in forest soil organic carbon transformation and turnover. Ecology and Evolution, 4, 633–647. https://doi.org/10.1002/ece3.969 25035803 10.1002/ece3.969 PMC4098142

[ece33708-bib-0061] You, Y. M. , Wang, J. , Sun, X. L. , Tang, Z. X. , Zhou, Z. Y. , & Sun, O. J. (2016). Differential controls on soil carbon density and mineralization among contrasting forest types in a temperate forest ecosystem. Scientific Reports, 6, 22411 https://doi.org/10.1038/srep22411 26925871 10.1038/srep22411 PMC4772072

[ece33708-bib-0062] Zhou, Z. , Jiang, L. , Du, E. Z. , Hu, H. F. , Li, Y. D. , Chen, D. X. , & Fang, J. Y. (2013). Temperature and substrate availability regulate soil respiration in the tropical mountain rainforests, Hainan Island, China. Journal of Plant Ecology, 6, 325–334. https://doi.org/10.1093/jpe/rtt034

